# Early-Onset Atrial Fibrillation and the Prevalence of Rare Variants in Cardiomyopathy and Arrhythmia Genes

**DOI:** 10.1001/jamacardio.2021.3370

**Published:** 2021-09-08

**Authors:** Zachary T. Yoneda, Katherine C. Anderson, Joseph A. Quintana, Matthew J. O’Neill, Richard A. Sims, Andrew M. Glazer, Christian M. Shaffer, Diane M. Crawford, Thomas Stricker, Fei Ye, Quinn Wells, Lynne W. Stevenson, Gregory F. Michaud, Dawood Darbar, Steven A. Lubitz, Patrick T. Ellinor, Dan M. Roden, M. Benjamin Shoemaker

**Affiliations:** 1 Division of Cardiovascular Medicine, Department of Medicine, Vanderbilt University Medical Center, Nashville, Tennessee; 2 Vanderbilt University School of Medicine, Nashville, Tennessee; 3 Division of Clinical Pharmacology, Department of Medicine, Vanderbilt University Medical Center, Nashville, Tennessee; 4 Department of Pathology, Microbiology and Immunology, Vanderbilt University Medical Center, Nashville, Tennessee; 5 Department of Biostatistics, Vanderbilt University Medical Center, Nashville, Tennessee; 6 Division of Cardiology, Department of Medicine, University of Illinois at Chicago, Chicago; 7 Cardiovascular Disease Initiative, The Broad Institute of MIT and Harvard, Cambridge, Massachusetts; 8 Cardiovascular Research Center, Massachusetts General Hospital, Boston; 9 Division of Clinical Pharmacology, Department of Medicine, Vanderbilt University Medical Center, Nashville, Tennessee; 10 Department of Pharmacology, Vanderbilt University Medical Center, Nashville, Tennessee; 11 Department of Biomedical Informatics, Vanderbilt University Medical Center, Nashville, Tennessee

## Abstract

**Importance:**

Early-onset atrial fibrillation (AF) can be the initial manifestation of a more serious underlying inherited cardiomyopathy or arrhythmia syndrome.

**Objective:**

To examine the results of genetic testing for early-onset AF.

**Design, Setting, and Participants:**

This prospective, observational cohort study enrolled participants from an academic medical center who had AF diagnosed before 66 years of age and underwent whole genome sequencing through the National Heart, Lung, and Blood Institute’s Trans-Omics for Precision Medicine program. Participants were enrolled from November 23, 1999, to June 2, 2015. Data analysis was performed from October 24, 2020, to March 11, 2021.

**Exposures:**

Rare variants identified in a panel of 145 genes that are included on cardiomyopathy and arrhythmia panels used by commercial clinical genetic testing laboratories.

**Main Outcomes and Measures:**

Sequencing data were analyzed using an automated process followed by manual review by a panel of independent, blinded reviewers. The primary outcome was classification of rare variants using American College of Medical Genetics and Genomics criteria: benign, likely benign, variant of undetermined significance, likely pathogenic, or pathogenic. Disease-associated variants were defined as pathogenic/likely pathogenic variants in genes associated with autosomal dominant or X-linked dominant disorders.

**Results:**

Among 1293 participants (934 [72.2%] male; median [interquartile range] age at enrollment, 56 [48-61] years; median [interquartile range] age at AF diagnosis, 50 [41-56] years), genetic testing identified 131 participants (10.1%) with a disease-associated variant, 812 (62.8%) with a variant of undetermined significance, 92 (7.1%) as heterozygous carriers for an autosomal recessive disorder, and 258 (20.0%) with no suspicious variant. The likelihood of a disease-associated variant was highest in participants with AF diagnosed before the age of 30 years (20 of 119 [16.8%; 95% CI, 10.0%-23.6%]) and lowest after the age of 60 years (8 of 112 [7.1%; 95% CI, 2.4%-11.9%]). Disease-associated variants were more often associated with inherited cardiomyopathy syndromes compared with inherited arrhythmias. The most common genes were *TTN* (n = 38), *MYH7* (n = 18), *MYH6* (n = 10), *LMNA* (n = 9), and *KCNQ1* (n = 8).

**Conclusions and Relevance:**

In this cohort study, genetic testing identified a disease-associated variant in 10% of patients with early-onset AF (the percentage was higher if diagnosed before the age of 30 years and lower if diagnosed after the age of 60 years). Most pathogenic/likely pathogenic variants are in genes associated with cardiomyopathy. These results support the use of genetic testing in early-onset AF.

## Key Points

**Question:**

In patients diagnosed with atrial fibrillation before 66 years of age, what is the prevalence of disease-associated variants in susceptibility genes for inherited cardiomyopathy and arrhythmia syndromes?

**Findings:**

In this cohort study, among 1293 participants who underwent whole genome sequencing, disease-associated rare variants in cardiomyopathy and arrhythmia genes were identified in 10.1% of participants younger than 66 years and 16.8% of those younger than 30 years. Disease-associated rare variants were more prevalent in genes associated with inherited cardiomyopathy syndromes than inherited arrhythmia syndromes.

**Meaning:**

The results of this study suggest that genetic testing in patients with early-onset atrial fibrillation identifies pathogenic variants associated with more serious inherited cardiomyopathy and arrhythmia syndromes.

## Teaser

This cohort study of individuals with early-onset atrial fibrillation examines the prevalence of disease-associated variants in susceptibility genes for inherited cardiomyopathy and arrhythmia syndromes.

## Introduction

Genetic testing is currently not recommended for atrial fibrillation (AF).^1,2^ However, recent data suggest that patients with early-onset AF are enriched for rare disease-associated variants, and case reports are emerging in which genetic testing for AF has changed clinical management.^3,4,5,6,7^ These data combined with increasing access to commercial genetic testing and inherited heart disease clinics have increased interest in genetic testing for AF, especially in younger patients and those with a strong family history of AF.^4^

Rare variants in genes associated with inherited arrhythmias (eg, long QT syndrome [LQTS]) and inherited cardiomyopathies (eg, hypertrophic cardiomyopathy [HCM]) have been known for decades to be associated with familial AF.^8,9,10^ More recently, rare loss-of-function variants in the *TTN* gene (OMIM 188840) have been found to be associated with AF in unselected patients with early-onset AF (defined as AF diagnosed before 66 years of age).^5^ Specifically, rare loss-of-function *TTN* variants were found in 2.1% of all patients with early-onset AF, and this proportion increased to 6.5% of patients diagnosed before the age of 30 years. In a subsequent report, 25 patients with early-onset AF underwent clinical genetic testing in an inherited heart disease clinic using a commercial arrhythmia and cardiomyopathy gene panel, and 6 (24%) carried a pathogenic or likely pathogenic (P/LP) variant in a clinically actionable gene.^4^ For context, this finding is comparable to the diagnostic yield of genetic testing in patients with dilated cardiomyopathy, which is approximately 25%,^1,11^ and greater than the rate of P/LP rare variants for clinically actionable genes in the general population, which is estimated to be approximately 2%.^12,13,14,15,16,17^

These results have led to a proposal that patients with early-onset AF be evaluated in an inherited heart disease clinic and, after appropriate genetic counseling, undergo genetic testing.^18^ This proposal represents a major change to the diagnostic workup for AF.^4,18^ However, many practical questions exist before implementing genetic testing for AF, such as what should be the age cutoff to consider genetic testing and what would be the yield of disease-associated variants. We report the results from 1293 participants with early-onset AF who underwent whole genome sequencing. We analyzed genes currently included on major commercial arrhythmia and cardiomyopathy gene panels to define the results according to clinical standards using the American College of Medical Genetics and Genomics (ACMG) classification and compare the frequency of disease-associated variants according to age at AF diagnosis, specific inherited syndromes, and individual genes.

## Methods

### Study Population

The study population were patients with early-onset AF (AF diagnosed before the age of 66 years) enrolled in the Vanderbilt Atrial Fibrillation or Vanderbilt AF Ablation Registries (see the eAppendix in the Supplement for description). Participants were enrolled from November 23, 1999, to June 2, 2015. Data analysis was performed from October 24, 2020, to March 11, 2021. All participants provided written informed consent, and participating studies obtained ethical approval from the Vanderbilt University Medical Center Institutional Review Board. Eligible participants underwent whole genome sequencing through the National Heart, Lung, and Blood Institute’s Trans-Omics for Precision Medicine (TOPMed) program as previously described.^5^ Race and ethnicity data were collected by participant self-report. Data submitted to TOPMed are deidentified. The study followed the Strengthening the Reporting of Observational Studies in Epidemiology (STROBE) reporting guideline.

### Whole Genome Sequencing

The sequencing methods and participant- and variant-level quality control steps for the TOPMed Atrial Fibrillation Project have been previously described^5^ and are available on the dbGaP website (https://www.ncbi.nlm.nih.gov/projects/gap/cgi-bin/study.cgi?study_id=phs001062.v5.p2).

### Selection of Genes for the Comprehensive Arrhythmia/Cardiomyopathy Panel

**Figure 1.**
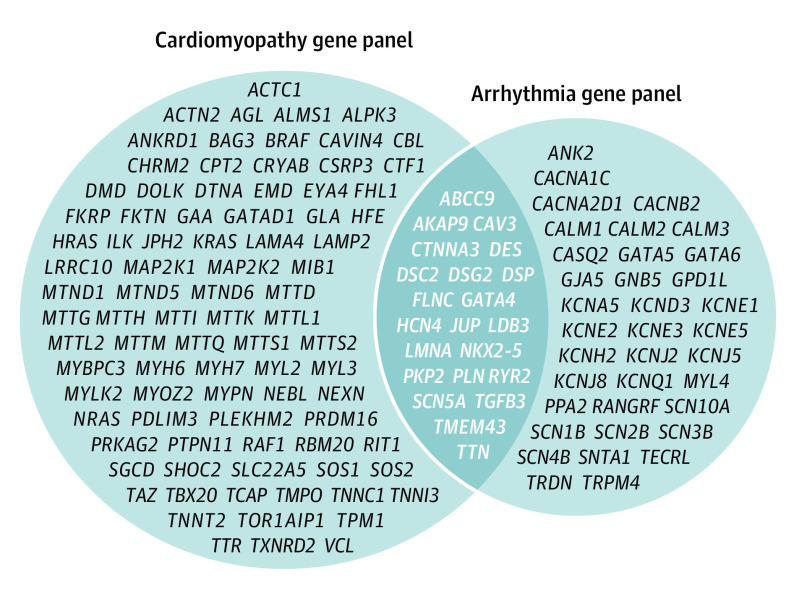
The Comprehensive Arrhythmia and Cardiomyopathy Gene Panel Genes were selected from commercial panels. A total of 145 genes were included; 87 were included only on the cardiomyopathy gene panel, 36 only on the arrhythmia gene panel, and 22 on both.

A major goal of this study was to simulate the clinical experience of genetic testing in patients with early-onset AF by using genes that matched those included on commercially available panels and to present results similar to those included on clinical genetic testing reports.^18^ Accordingly, we selected genes from the comprehensive cardiomyopathy and arrhythmia panels for 1 of several commercial genetic testing companies (Ambry Genetics, GeneDx, or Invitae Inc). Because commercial gene panels undergo continual review and revision, the gene panels in this study were current as of June 1, 2020. Figure 1 shows the 145 genes included and displays the overlap between those on both the arrhythmia and cardiomyopathy panels. To analyze the frequency for which genetic testing for early-onset AF may suggest an overlapping inherited arrhythmia or cardiomyopathy syndrome, genes for which a disease-associated variant was detected were assigned to a specific syndrome (if applicable): arrhythmogenic cardiomyopathy/arrhythmogenic right ventricular cardiomyopathy (AC/ARVC), Brugada syndrome, catecholaminergic polymorphic ventricular tachycardia (CPVT), dilated cardiomyopathy (DCM), HCM, and LQTS. Some genes are associated with multiple syndromes (eg, *SCN5A* [OMIM 600163] and *LMNA* [OMIM 150330]) and therefore were assigned to more than 1 (eTable 1 in the Supplement). Genes were assigned to a syndrome based on their status in the Clinical Genome Resource (ClinGen),^19^ and those classified as having strong or definitive evidence by ClinGen were labeled as *major disease genes*.

### Variant Annotation, Filtration, and Interpretation

Analysis was restricted to the 145 genes included on our panel. For variant prioritization and interpretation, an automated artificial intelligence–based process was used (Franklin, Genoox Ltd), which builds disease association and deleteriousness prediction models at the gene and variant levels by integrating information from multiple gene and variant classification sources (eg, ClinVar, ClinGen, Uniprot, and gnomAD).^20^ The automated algorithm classifies each variant according to ACMG criteria into the following categories: benign (B), likely benign (LB), variant of undetermined significance (VUS), likely pathogenic (LP), and pathogenic (P).^21^ The VUS category is further subdivided into VUS–possibly benign (VUS-PB), VUS-uncertain (VUS-U), and VUS–possibly pathogenic (VUS-PP) by considering the results from a variety of in silico prediction tools.^22^ Following automated ACMG classification, rare variants were categorized as B, LB, VUS, LP, or P. Next, all P/LP variants and VUS-PP were manually reviewed to reassess pathogenicity, which included verifying that ACMG criteria were appropriately applied, such as the allele frequency (PM2), reputable source criteria (PP5),^23^ confirming loss of function was a known disease mechanism for a given gene (PVS1),^24^ and searching the literature for new publications (eFigure 1 in the Supplement). This process was performed by 2 independent, blinded reviewers with expertise in clinical cardiogenetics (M.B.S. and K.C.A.), and disagreements were settled by a third independent reviewer (A.M.G.). Statistics for interobserver agreement are reported in the Results section.

### Statistical Analysis

Results are reported at the variant and participant levels. Count variables are presented as number (percentage). Point estimates with 95% CIs were generated for all proportions via 10 000 bootstrapped samples with replacement. For continuous variables, medians (interquartile ranges [IQRs]) are reported. Univariable logistic regression models were used to assess the association between variant detection and age, sex, race, and ethnicity. To assess possible nonlinear associations of age on hazard, a restricted cubic spline function was used with 3 knots. An *F* test was used to test against the hypothesis that all age terms in the restricted cubic spline function were 0 as well as to evaluate for nonlinearity. To evaluate the independent association of age on variant detection, a multivariable logistic regression model was fitted with adjustment for sex, race, and ethnicity. The Cohen κ coefficient measured interobserver agreement for the variant reviewers. Statistical analyses used R, version 4.0.0 (R Foundation for Statistical Computing) and Stata, version 16 (StataCorp LLC). GraphPad Prism, version 5.04 (GraphPad Software) was used for figures. A 2-sided *P* < .05 was considered statistically significant.

## Results

### Description of the Study Population

The Table gives the clinical characteristics at the time of enrollment for the 1293 participants (934 [72.2%] male; median [IQR] age at enrollment, 56 [48-61] years; median [IQR] age at AF diagnosis, 50 [41-56] years) included in this study. No participants were excluded. The study cohort included 1238 White participants (95.7%), 48 Black participants (3.7%), and 7 participants (0.5%) of other races (6 Asian participants [0.5%] and 1 Native American/Alaskan Native participant [0.1%]); ethnicity included 1286 non-Hispanic participants (99.5%) and 7 Hispanic participants (0.5%). The Table presents data stratified by rare variant status. Participants with disease-associated variants were more likely to have a history of heart failure (36 [27.5%]) compared with the other groups (*P* = .001). When examined separately, heart failure with reduced ejection fraction (15.3% in group 1, *P* = .002) was also significantly higher. Heart failure with preserved ejection fraction was higher (12.2% in group 1, *P* = .16), although not statistically significant. These results suggest disease-associated cardiomyopathy variants confer a genetic susceptibility to left ventricular dysfunction, and future studies will seek to define its temporal association with the initial onset of AF. When group 2 participants were restricted to only those with VUS-PP, rates were 13.7% for heart failure, 8.0% for heart failure with reduced ejection fraction, and 5.7% for heart failure with preserved ejection fraction, which is comparable to participants with no suspicious variants.

**Table.** Demographic and Baseline Clinical Characteristics^a^

Characteristic	Overall (N = 1293)	Group 1 (disease-associated, variant) (n = 131)	Group 2 (VUS) (n = 812)	Group 3 (carrier for AR disorder (n = 92)	Group 4 (no suspicious variant) (n = 258)
Age at enrollment, y					
Median (IQR)	56 (48-61)	53 (43-59)	56 (49-61)	55 (45-60.5)	56 (49-61)
<30	52 (4.0)	12 (9.2)	31 (3.8)	1 (1.1)	8 (3.1)
30-39	96 (7.4)	11 (8.4)	60 (7.4)	13 (14.1)	12 (4.7)
40-49	225 (17.4)	32 (24.4)	131 (16.1)	17 (18.5)	45 (17.4)
50-59	521 (40.3)	46 (35.1)	328 (40.4)	33 (35.9)	114 (44.2)
60-65	399 (30.9)	30 (22.9)	262 (32.3)	28 (30.4)	79 (30.6)
Age at AF diagnosis, y					
Median (IQR)	50 (41-56)	48 (39-56)	50 (42-56)	49 (38.5-55)	50 (44-55)
<30	119 (9.2)	20 (15.3)	76 (9.4)	10 (10.9)	13 (5.0)
30-39	143 (11.1)	15 (11.5)	90 (11.1)	14 (15.2)	24 (9.3)
40-49	364 (28.2)	36 (27.5)	218 (26.9)	23 (25.0)	87 (33.7)
50-59	555 (42.9)	52 (39.7)	346 (42.6)	38 (41.3)	119 (42.9)
60-65	112 (8.7)	8 (6.1)	82 (10.1)	7 (7.6)	15 (5.8)
Sex					
Male	934 (72.2)	89 (67.9)	594 (73.2)	66 (71.7)	185 (71.7)
Female	359 (27.8)	42 (32.1)	218 (26.9)	26 (28.3)	73 (28.3)
Self-reported race					
White	1238 (95.7)	127 (97.0)	768 (94.6)	92 (100.0)	251 (97.3)
Black	48 (3.7)	3 (2.3)	39 (4.8)	0 (0)	6 (2.3)
Other	7 (0.5)	1 (0.8)	5 (0.6)	0 (0)	1 (0.4)
Self-reported ethnicity					
Non-Hispanic	1286 (99.5)	130 (99.2)	809 (99.6)	92 (100.0)	255 (98.8)
Hispanic	7 (0.5)	1 (0.8)	3 (0.4)	0 (0.0)	3 (1.2)
Height, median (IQR), cm	178 (170-185)	178 (170-185)	178 (170-185)	179 (170-183)	180 (170-185)
BMI					
Median (IQR)	30.2 (26.6-35.2)	30.1 (25.8-34.1)	30.4 (26.6-35.6)	30.4 (27.2-34.3)	30.0 (26.5-34.8)
≥30	652 (50.4)	65 (51.2)	412 (50.7)	46 (50.0)	129 (50.0)
Obstructive sleep apnea	236 (18.3)	19 (14.5)	156 (19.3)	17 (18.5)	44 (17.1)
Hypertension	729 (56.4)	69 (52.7)	475 (58.5)	50 (54.4)	135 (52.3)
Valve disease	96 (7.5)	14 (10.7)	60 (7.4)	4 (4.4)	18 (7.0)
Myocardial infarction	92 (7.1)	6 (4.6)	62 (7.6)	7 (7.6)	17 (6.6)
Heart failure	221 (17.1)	36 (27.5)	126 (15.5)	19 (20.7)	40 (15.5)
Reduced ejection fraction	106 (8.2)	20 (15.3)	59 (7.3)	10 (10.9)	17 (6.6)
Preserved ejection fraction	115 (8.9)	16 (12.2)	67 (8.2)	9 (9.8)	23 (8.9)
Left ventricular ejection fraction, %^b^					
Median (IQR)	55 (53-60)	55 (50-60)	55 (54-60)	55 (52-61)	55 (54-60)
<40	106 (8.5)	15 (12.0)	59 (7.6)	12 (13.3)	20 (8.0)
40-49	87 (7.0)	10 (8.0)	51 (6.6)	8 (8.9)	18 (7.2)
≥50	1049 (84.5)	100 (80.0)	666 (85.8)	70 (77.8)	213 (84.9)

Abbreviations: AF, atrial fibrillation; AR, autosomal recessive; BMI, body mass index (calculated as weight in kilograms divided by height in meters squared); IQR, interquartile range; VUS, variant of undetermined significance.

^a^ Data are presented as number (percentage) of participants unless otherwise indicated.

^b^ Left ventricular ejection fraction as measured by echocardiography. Echocardiograms were missing from a total of 51 participants (3.9%) evenly distributed among the groups: 6 (4%) in group 1, 36 (4%) in group 2, 2 (2%) in group 3, and 7 (3%) in group 4.

### Results of ACMG Variant Classification

**Figure 2.**
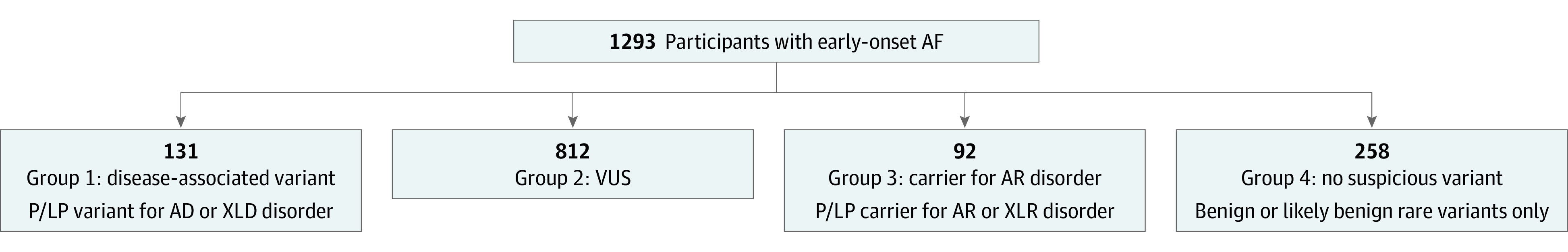
Results of Genetic Testing in Early-Onset Atrial Fibrillation (AF) for Genes Associated With Arrhythmia and Cardiomyopathy Syndromes Variants are classified according to standard American College of Medical Genetics and Genomics criteria. AD indicates autosomal dominant; AR, autosomal recessive; P/LP, pathogenic/likely pathogenic; VUS, variant of undetermined significance; XLD, X-linked dominant; XLR, X-linked recessive.

Eligible participants were placed in mutually exclusive groups according to variant status (Figure 2). Group assignment was based on a participant’s highest priority variant, with group 1 as the highest priority and group 4 as the lowest. Group 1 participants carried at least 1 P/LP rare variant in a gene associated with an autosomal dominant or X-linked dominant disorder (in men). Group 1 participants were considered to be carrying a disease-associated variant and comprised 131 members (10.1%) of the study cohort. Qualifying variants for the group 1 participants are listed in eTables 2 and 3 in the Supplement. Nine participants had 2 or more group 1 variants and are presented in eTable 4 in the Supplement. Group 2 participants carried a VUS and were not included in group 1. Group 2 comprised 812 (62.8%) of the cohort. Group 3 participants were heterozygous for a P/LP variant in a gene associated with an autosomal recessive or X-linked recessive disorder. Group 3 participants were considered to be carriers for an autosomal recessive disorder and comprised 92 members (7.1%) of the study cohort. A total of 82 participants (6.3%) in group 3 carried a variant in *HFE* (OMIM 613609), the disease gene for hemochromatosis. Group 4 participants carried no P/LP variants or VUSs and comprised 258 members (20.0%) of the cohort. For the manual review, interobserver agreement was 91.8% (κ coefficient = 0.848).

### Prevalence of Disease-Associated Rare Variants Stratified by Age

**Figure 3.**
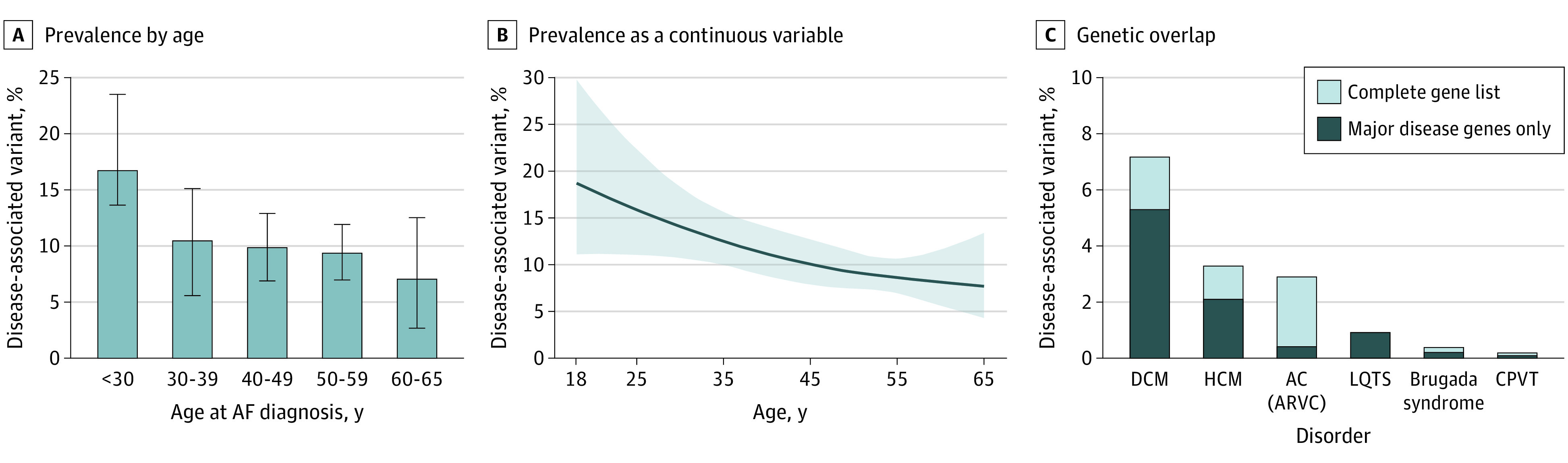
Prevalence of Disease-Associated Variants and Genetic Overlap With Inherited Cardiomyopathy and Arrhythmia Syndromes A, Prevalence of disease-associated rare variants according to age at atrial fibrillation (AF) diagnosis presented by age groups. Error bars indicate bootstrapped 95% CIs. B, Prevalence of disease-associated rare variants presented as a continuous variable (cubic spline graph, *P* = .02 for the association between age and presence of disease-associated variant based on the *F* test). C, The genetic overlap between disease-associated variants and specific inherited cardiomyopathy and arrhythmia syndromes. Shaded in blue is the proportion of variants in major disease genes for each disorder. AC (ARVC) indicates arrhythmogenic cardiomyopathy (arrhythmogenic right ventricular cardiomyopathy); CPVT, catecholaminergic polymorphic ventricular tachycardia; DCM, dilated cardiomyopathy; HCM, hypertrophic cardiomyopathy; LQTS, long QT syndrome.

The number of disease-associated variants was highest among participants diagnosed with AF before the age of 30 years (20 of 119 [16.8%]; 95% CI, 10.1%-23.5%) (Figure 3A). The numbers among the other age groups were 15 of 143 (10.5%; 95% CI, 5.6%-16.1%) among those 30 to 39 years of age, 36 of 364 (9.9%; 95% CI, 6.9%-12.9%) among those 40 to 49 years of age, 52 of 555 (9.4%; 95% CI, 7.0%-11.9%) among those 50 to 50 years of age, and 8 of 112 (7.1%; 95% CI, 2.7%-12.5%) among those 60 to 65 years of age. In univariate analysis, younger age significantly increased the likelihood of detecting a disease-associated variant; the odds increased by 1.25 per decade of earlier diagnosis (95% CI, 1.06-1.47; *P* = .007). The likelihood of detecting a disease-associated rare variant using a nonlinear age term is presented in Figure 3B. Results with multivariable adjustment for sex, race, and ethnicity were similar to the univariate analysis (odds ratio, 1.26 per decade of earlier diagnosis; 95% CI, 1.07-1.48; *P* = .005). No association was found between age at AF diagnosis and being in the VUS-only group (group 2) (odds ratio, 1.01; 95% CI, 1.00-1.02; *P* = .27).

### Genetic Overlap With Other Inherited Arrhythmia and Cardiomyopathy Syndromes

Disease-associated rare variants (group 1) were more prevalent in genes associated with inherited cardiomyopathy syndromes than inherited arrhythmia syndromes (Figure 3C). Specifically, the numbers of participants with a disease-associated rare variant were 93 (7.2%) for DCM, 43 (3.3%) for HCM, and 37 (2.9%) for AC/ARVC. These findings compare to lower rates for inherited arrhythmias: 2 (0.2%) for Brugada syndrome, 12 (0.9%) for LQTS, and 1 (0.1%) for CPVT. When restricted to major disease genes, numbers were 69 (5.3%) for DCM, 27 (2.1%) for HCM, 5 (0.4%) for AC/ARVC, 2 (0.2%) for Brugada syndrome, 11 (0.9%) for LQTS, and 1 (0.1%) for CPVT.

### Prevalence of Rare Variants in Specific Genes

**Figure 4.**
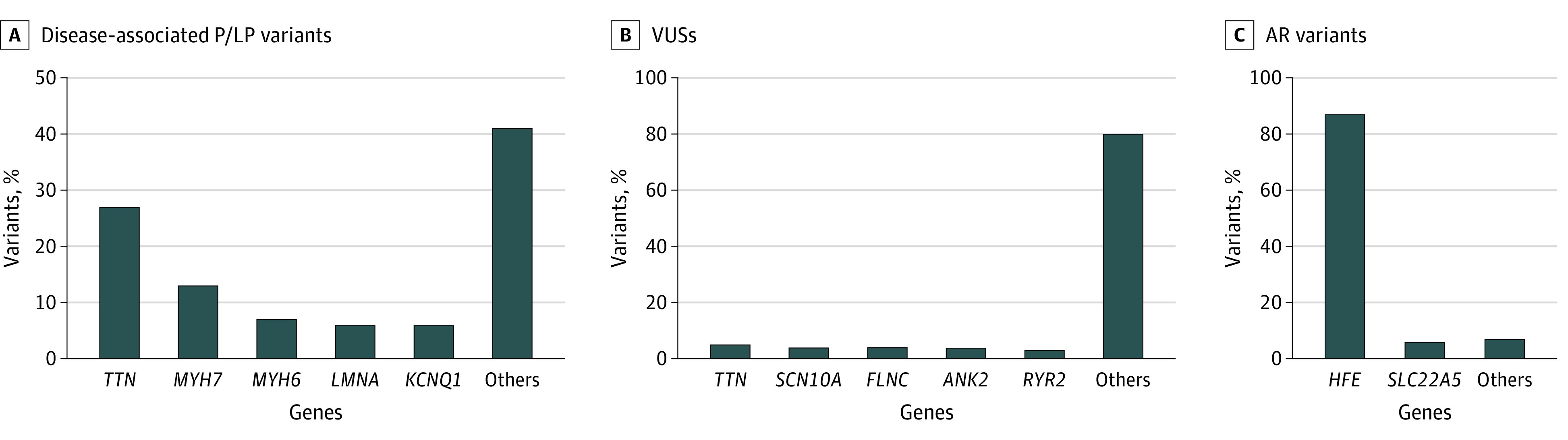
Breakdown According to the Most Prevalent Genes A, Pathogenic/likely pathogenic (P/LP) variants in autosomal dominant disorders. B, Variants of undetermined significance (VUSs); only loss-of-function variants in *TTN* are reported. C, Heterozygous P/LP variants in autosomal recessive (AR) disorders.

There were 141 P/LP variants for AD or X-linked dominant disorders in 34 different genes (Figure 4A). For the genes with the most prevalent group 1 variants, there were 38 (27%) in *TTN*, 18 (13%) in *MYH7* (OMIM 160760), 9 (6%) in *LMNA*, 10 (7%) in *MYH6* (OMIM 160710), and 8 (6%) in *KCNQ1* (OMIM 607542). Ages at AF diagnosis were as follows: 44 years (IQR, 36-55 years) for *TTN*, 48 years (IQR, 29-53 years) for *MYH7*, 43 years (IQR, 36-56 years) for *MYH6*, 52 years (IQR, 41-52 years) for *LMNA*, and 43 years (IQR, 29-57 years) for *KCNQ1*. There were 1979 VUSs in 104 different genes (Figure 4B), and 812 participants (62.8%) had a VUS alone. The VUSs in *TTN* were the most prevalent (n = 98 loss-of-function variants). Rare missense variants in *TTN* were not reported (n = 494 in our cohort). The VUSs were also common in *SCN10A* (OMIM 604427) (n = 86 in our cohort). Other common VUSs were in *RYR2* (OMIM 180902) (n = 66) and *FLNC* (OMIM 102565) (n = 81). A breakdown of VUS-PP per gene is separately reported in eFigure 2A in the Supplement. The age at AF diagnosis for participants with a VUS-PP was 52 years (IQR, 44-57 years). With the use of linear regression, the number of VUSs per individual was significantly associated with transcript length (β = 3.36 VUSs per kilobase pair; 95% CI, 2.61-4.11 kilobase pair; *P* = .001). When restricted to the VUS-PP subgroup, the association becomes weaker (β = 0.30 VUS-PPs per kilobase pair; 95% CI, 0.02-0.58 kilobase pair; *P* = .03). With the use of the linear regression model to predict the number of VUS-PPs, the numbers observed are greater than predicted for the most prevalent genes (eFigure 2B in the Supplement). There were 520 P/LP variants for AR or X-linked recessive disorders in 11 genes (Figure 4C). Variants in the gene *HFE* (homeostatic iron regulator), which cause hemochromatosis, accounted for 87% of variants in this category. A total of 24 participants had 2 P/LP rare variants in *HFE*, and 1 of them was diagnosed with hemochromatosis. Among participants with 2 *HFE* variants, the data were not available to determine whether the variants were on the same (cis) or different (trans) alleles. No participants had 2 P/LP rare variants in any other genes.

## Discussion

In this cohort study, we analyzed sequencing data from 1293 patients with early-onset AF for genes included on currently available commercial arrhythmia and cardiomyopathy gene panels and used methods for variant classification and reporting that simulate those used in clinical practice. Recent evidence^4,18^ has led to clinical genetic testing being considered for select patients with early-onset AF using comprehensive arrhythmia and cardiomyopathy gene panels. However, data are currently limited to inform practitioners and genetic counselors about the expected results. Our results found that the overall yield of positive genetic test results for disease-associated variants was 10.1% for patients diagnosed with AF before 66 years of age and up to 16.8% in patients diagnosed before 30 years of age. When a disease-associated variant is detected, additional diagnostic evaluation or gene-guided management is recommended for genetic overlap syndromes,^18^ which our results found is greater between early-onset AF and inherited cardiomyopathy syndromes (DCM, HCM, and AC/ARVC) than channelopathies (LQTS and Brugada syndrome). As more clinical practice documents have incorporated genotype into the diagnostic and management algorithms for inherited cardiomyopathies and arrhythmias,^25,26^ cases in which genetic testing for early-onset AF has changed clinical management are emerging.^3^

### Age-Related Prevalence of Disease-Associated Variants

It is currently unknown what the recommended age cutoff should be to consider genetic testing for AF. We previously proposed that it should be patients diagnosed before 45 years of age.^18^ However, our results demonstrate that the likelihood of a disease-associated variant is approximately the same (10%) in the 40- to 49- and 50- to 59-year age groups, with a decrease after 60 years of age. These data suggest that genetic testing for early-onset AF could be considered in patients diagnosed with AF up to 60 years of age, with a stronger recommendation for patients diagnosed before 30 years of age.

### Genetic Overlap Between Early-Onset AF and Inherited Arrhythmias and Cardiomyopathies

Our results suggest a high degree of genetic overlap between early-onset AF and inherited cardiomyopathy syndromes and, to a lesser degree, inherited arrhythmia syndromes. This finding is consistent with prior results that found that 28.8% of patients with an inherited cardiomyopathy syndrome had AF compared with 8.2% of patients with an inherited arrhythmia syndrome.^27^ We found that disease-associated variants were most frequent in genes associated with DCM followed by AC/ARVC and HCM. There is considerable overlap among the genetic causes of DCM, AC/ARVC, and HCM, and collectively these patients may represent a genetic subtype of AF characterized by the early development of an atrial myopathy.^7,28^ An important future question is to what degree patients with early-onset AF attributable to a cardiomyopathy-associated variant will develop heart failure, ventricular arrhythmias, and stroke and whether early identification may present the opportunity to modify the progression of disease and indicate the need for more aggressive control of traditional clinical risk factors.

### Prevalence of Disease-Associated Variants in Individual Genes

Consistent with prior reports,^4,5,7,29^ we found that loss-of-function variants in *TTN* were the most commonly associated variants in early-onset AF (27% of disease-associated variants) (Figure 4A). The next most common was *MYH7* (13%), encoding β-myosin heavy chain, which supports prior observations that patients with HCM attributable to *MYH7* variants have a significantly higher risk of AF than those with variants in other sarcomeric genes.^27^ Further supporting the potential importance of myosin heavy chain subunits on atrial structure and function, variants in *MYH6*, which encodes the α-subunit predominantly expressed in atrium, were also common (7%).^30,31^ Other top genes were *LMNA* (6%), which encodes lamin A and C and is responsible for an especially arrhythmogenic form of DCM with early-onset conduction disease, ventricular tachycardia, and AF,^32^ and *KCNQ1* (6%), which causes type 1 LQTS.^8,33^

### Variants of Undetermined Significance

Variants of undetermined significance represent a major challenge in genetic testing because they comprise a large proportion of the total variants reported. Our results demonstrate that this is true for early-onset AF. Nearly two-thirds of participants in our cohort had a VUS alone. The VUSs in *TTN* were the most prevalent, despite only loss-of-function variants in *TTN* being reported. Missense variants in *TTN* are often not reported by commercial genetic testing laboratories because they are extremely common and their clinical relevance has not been established.^34^ Variants of undetermined significance were also common in *SCN10A*. Intronic single-nucleotide polymorphisms within *SCN10A* have been identified by genome-wide association studies to be strongly associated with AF and cardiac conduction; however, the mechanism of that association and the potential role of rare variants within *SCN10A* remain to be elucidated.^35,36,37,38,39,40,41^ Other common VUSs were found in *RYR2*, which is the most common cause of CPVT,^42,43^ and *FLNC*, which is a common cause of AC and DCM.^44^ Both *RYR2* and *FLNC* are associated with potentially fatal ventricular arrhythmias and are examples of why genetic counseling with anticipatory guidance may be especially useful to address anxiety that may be caused by detecting a VUS.^44,45^

### Limitations

This study is subject to the limitations that currently affect clinical genetic testing, including disagreement on ACMG classification for a given variant.^46^ In addition, gene curation efforts are ongoing, and many genes included on commercial panels have varying levels of evidence for their association with specific cardiac phenotypes, and specific gene panels for AF have not been developed. The study population was composed predominately of people of European ancestry. A recent report^47^ indicated that 7% of patients of African and Hispanic descent with early-onset AF who underwent sequencing using a 60-gene panel possessed a P/LP rare variant, and those variants in *TTN* were the most common. These data begin to define the prevalence of rare variants associated with AF in individuals of underrepresented ethnicities; however, more research is needed. In addition, because this was a single-center study, enrichment for genetic causes of AF may vary, depending on differences in referral patterns and patient populations among medical centers.

## Conclusions

Genetic testing in patients with early-onset AF for genes included on commercial arrhythmia and cardiomyopathy panels detected a disease-associated variant in approximately 10% of patients diagnosed with early-onset AF. The rate was higher in patients diagnosed before the age of 30 years and lower in those diagnosed after 60 years of age. Disease-associated variants were more common in genes associated with cardiomyopathies than channelopathies, and the most affected genes were *TTN, MYH7, MYH6, LMNA,* and *KCNQ1.* The results of this study help to inform decisions regarding genetic testing in patients presenting with early-onset AF.

## Notes

Supplement. **eAppendix.** Description of Parent Studies **eFigure 1.** Variant Classification Process **eFigure 2.** Breakdown According to the Most Prevalent Genes for VUS-Possibly Pathogenic (VUS-PP) (A) and the Difference Between the Number of VUS-PP Observed vs the Number of VUS-PP Predicted Based on Gene Length (B) **eTable 1.** Genes For Which Disease-Associated Variants Were Identified and Assigned to Specific Inherited Cardiomyopathy and Arrhythmia Syndromes **eTable 2.** List of P/LP Rare Variants (Except *TTN* Variants) **eTable 3.** List of P/LP Loss-pf-Function *TTN* Variants **eTable 4.** List of Participants With 2 or More Disease-Associated Variants
