# Supplementary material for: Early-Onset Atrial Fibrillation and the Prevalence of Rare Variants in Cardiomyopathy and Arrhythmia Genes
Source: JAMA Cardiol. 2021 Sep 8;6(12):1–9. doi: 10.1001/jamacardio.2021.3370 (PMC8427496; doi:10.1001/jamacardio.2021.3370)
Supplement: Supplement. — eAppendix. Description of Parent Studies eFigure 1. Variant Classification Process eFigure 2. Breakdown According to the Most Prevalent Genes for VUS-Possibly Pathogenic (VUS-PP) (A) and the Difference Between the Number of VUS-PP Observed vs the Number of VUS-PP Predicted Based on Gene Length (B) eTable 1. Genes For Which Disease-Associated Variants Were Identified and Assigned to Specific Inherited Cardiomyopathy and Arrhythmia Syndromes eTable 2. List of P/LP Rare Variants (Except TTN Variants) eTable 3. List of P/LP Loss-pf-Function TTN Variants eTable 4. List of Participants With 2 or More Disease-Associated Variants [file jamacardiol-e213370-s001.pdf]

Supplementary Online Content

Yoneda ZT, Anderson KC, Quintana JA, et al. Early-onset atrial fibrillation and the prevalence of rare variants in cardiomyopathy and arrhythmia genes. *JAMA Cardiol.* Published online September 8, 2021. doi:10.1001/jamacardio.2021.3370

eAppendix. Description of Parent Studies

eFigure 1. Variant Classification Process

eFigure 2. Breakdown According to the Most Prevalent Genes for VUS-Possibly Pathogenic (VUS-PP) (A) and the Difference Between the Number of VUS-PP Observed vs the Number of VUS-PP Predicted Based on Gene Length (B)

eTable 1. Genes For Which Disease-Associated Variants Were Identified and Assigned to Specific Inherited Cardiomyopathy and Arrhythmia Syndromes

eTable 2. List of P/LP Rare Variants (Except *TTN* Variants)

eTable 3. List of P/LP Loss-pf-Function *TTN* Variants

eTable 4. List of Participants With 2 or More Disease-Associated Variants

This supplementary material has been provided by the authors to give readers additional information about their work.

© 2021 Yoneda ZT et al. *JAMA Cardiol.*

eAppendix. Description of parent studies

Vanderbilt Atrial Fibrillation Registry: The Vanderbilt Atrial Fibrillation (AF) Registry was started in

2001. It is approved by the Vanderbilt University Medical Center Institutional Review Board (IRB # 020669; PI: Roden). All participants undergo written, informed consent. Patients with AF and family members are prospectively enrolled. At enrollment, a detailed past medical history is obtained along with an AF symptom severity assessment. Blood samples are obtained for DNA extraction. Patients are followed longitudinally to track clinic outcomes.

The Vanderbilt Atrial Fibrillation Ablation Registry (VAFAR) was started in 2011. It is approved by the Vanderbilt University Medical Center Institutional Review Board (IRB #110881; PI: Shoemaker). All participants undergo written, informed consent. It is a prospective observational registry of subjects undergoing AF ablation (clinicaltrials.gov NCT #02404415). Whole blood is collected during the ablation procedure from which DNA is extracted and stored along with serum and plasma. Baseline clinical data are manually extracted from the medical record and supplemented by patient interview. Pre-ablation imaging studies are performed (cardiac MRI or CT) and stored. Electrophysiologic data obtained at the time of ablation is collected and stored (data from the baseline electrophysiologic study and data and images from the electroanatomic map). Participants are prospectively followed for arrhythmia recurrence post-ablation and longitudinally to track longer term clinical outcomes.

© 2021 Yoneda ZT et al. *JAMA Cardiol.*

eFigure 1. Variant classification process

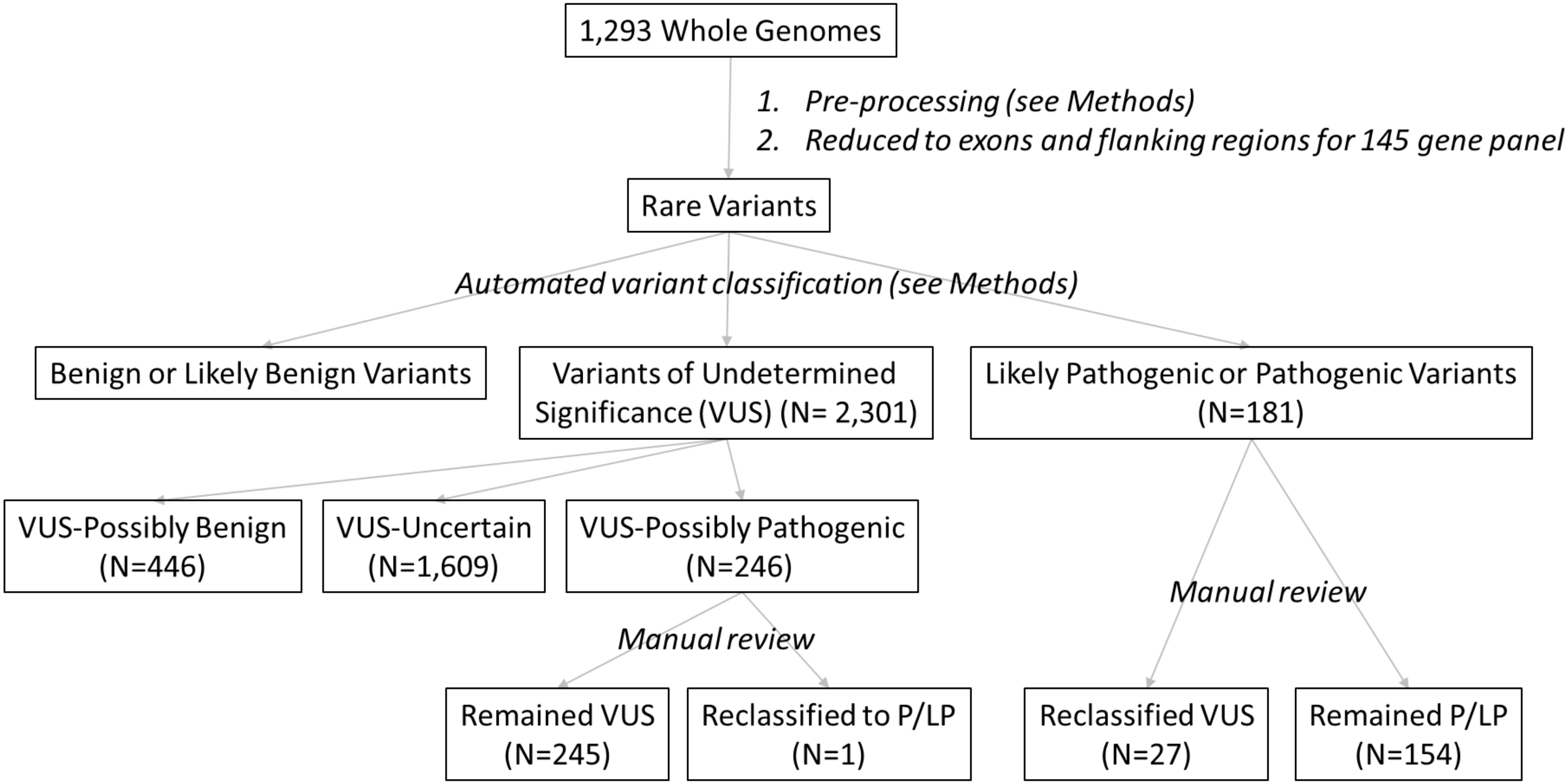

© 2021 Yoneda ZT et al. *JAMA Cardiol.*

eFigure 2. Breakdown According to the Most Prevalent Genes for VUS-Possibly Pathogenic (VUS-PP) (A) and the Difference Between the Number of VUS-PP Observed vs the Number of VUS-PP Predicted Based on Gene Length (B)

Data are unadjusted for transcript length. **TTN* loss-of-function variants only. A linear regression was fit to the observed data (Panel A) using transcript length as the sole independent predictor (β= 0.30 VUS-PP/kbp [95% CI: 0.02-0.58, P= 0.03]). Predicted VUS-PP by this linear regression model are reported in the third column, with difference between observed and expected reported in column four.

A.

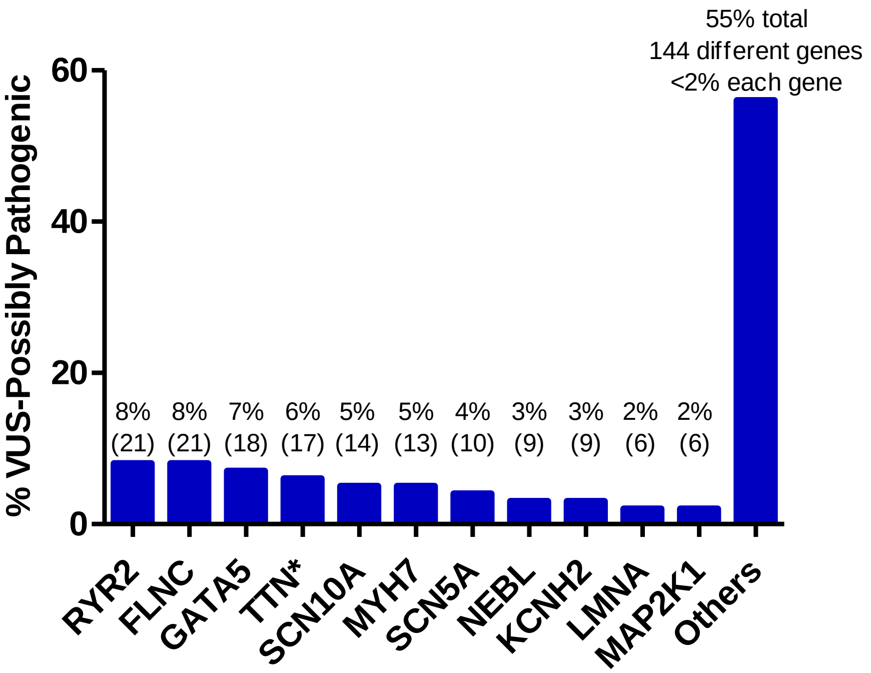

B.

	**Number of VUS-PP**		
	**Observed**	**Predicted by transcript length***	**Difference**
RYR2	21	7	+14
FLNC	21	4	+17
GATA5	18	2	+16
*TTN**	N/A	N/A	N/A
SCN10A	14	4	+10
MYH7	15	3	+11
SCN5A	10	4	+6
NEBL	9	4	+5
KCNH2	9	3	+6
LMNA	6	3	+3
MAP2K1	6	2	+4

*Using a linear regression model based on gene length.
N/A: *TTN* is not analyzed because only loss-of-function variants are reported.

© 2021 Yoneda ZT et al. *JAMA Cardiol.*

eTable 1. Genes for which disease-associated variants were identified and assigned to overlapping inherited cardiomyopathy and arrhythmia syndromes

**ARVC/AC**		**Brugada**	**CPVT**	**DCM**			**HCM**		**LQTS**
DES	*MYL3*	*SCN5A*	*CASQ2*	*ACTC1*	*LMNA*	*SCN5A*	*ACTC1*	*MYL3*	*CACNA1C*
DSP	*SCN5A*			*CSRP3*	*MYBPC3*	*TPM1*	*CSRP3*	*TNNT2*	*KCNH2*
FLNC	*TTN*			*DES*	*MYH6*	*TTN*	*FHL1*	*TPM1*	*KCNQ1*
JUP				*DSP*	*MYH7*	*VCL*	*MYBPC3*	*TTN*	*SCN5A*
LMNA				*FHL1*	*NKX2-5*		*MYH6*	*VCL*	
MYH7				*FLNC*	*TNNT2*		*MYH7*		

*Strong or definite disease genes as currently defined by ClinGen (https://www.clinicalgenome.org) are shaded. Genes not shaded have lesser evidence.

© 2021 Yoneda ZT et al. *JAMA Cardiol.*

eTable 2. List of rare variants classified as disease-associated (except *TTN* variants)

Variant (DNA)	Variant (protein)	Gene	Mutation Type	Race/ Ethnicity	Variant Allele Count gnomAD*	Total Allele Number gnomAD	Inheritance	ACMG Class
c.56_57insCA	p.Lys20fs	*ACTC1*	Frameshift	White/NH	2	31378	AD	LP
c.941G>A	p.Arg314His	*ACTC1*	Missense	White/NH	4	282300	AD	LP
c.7081C>T	p.Gln2361X	*AKAP9*	Nonsense	White/NH	1	31402	AD	LP
c.2161_2165delATCAA	p.Ile721fs	*AKAP9*	Frameshift	White/NH	2	264486	AD	LP
c.7977_7978delGA	p.Lys2660fs	*AKAP9*	Frameshift	White/NH	2	255474	AD	LP
c.5977C>T	p.Gln1993X	*AKAP9*	Nonsense	White/NH	2	280500	AD	LP
c.1364G>A	p.Gly455Glu	*BRAF*	Missense	White/NH	1	31404	AD	LP
c.722C>T	p.Thr241Met	*BRAF*	Missense	White/NH	1	279894	AD	P
c.15_16delTA	p.Thr6fs	*CACNA1C*	Frameshift	White/NH	3	257862	AD	LP
c.923C>T	p.Pro308Leu	*CASQ2*	Missense	White/NH	9	282770	AD	P
c.338C>T	p.Ser113Leu	*CPT2*	Missense	White/NH	393	282834	AD	P
c.338C>T	p.Ser113Leu	*CPT2*	Missense	White/NH	393	282834	AD	P
c.338C>T	p.Ser113Leu	*CPT2*	Missense	White/NH	393	282834	AD	P
c.338C>T	p.Ser113Leu	*CPT2*	Missense	White/NH	393	282834	AD	P
c.1891C>T	p.Arg631Cys	*CPT2*	Missense	White/NH	7	282810	AD	LP
c.338C>T	p.Ser113Leu	*CPT2*	Missense	White/NH	393	282834	AD	P
c.338C>T	p.Ser113Leu	*CPT2*	Missense	White/NH	393	282834	AD	P
c.131T>C	p.Leu44Pro	*CSRP3*	Missense	White/NH	4	282276	AD	LP
c.415-1G>T	N/A	*CSRP3*	Splice Site	White/NH	2	282724	AD	LP
c.449G>A	p.Cys150Tyr	*CSRP3*	Missense	White/NH	4	282834	AD	LP
c.449G>A	p.Cys150Tyr	*CSRP3*	Missense	White/NH	4	282834	AD	LP
c.1288+1G>A	N/A	*DES*	Splice Site	White/NH	6	282842	AD	LP
c.3751delG	p.Asp1251fs	*DSP*	Frameshift	White/NH	1	31406	AD	LP
c.5671_5674delGAGA	p.Glu1891fs	*DSP*	Frameshift	White/NH	1	30740	AD	LP
c.5671_5674delGAGA	p.Glu1891fs	*DSP*	Frameshift	White/NH	1	30740	AD	LP
c.542G>A	p.Trp181X	*FHL1*	Nonsense	White/NH	1	21776	XLR	LP
c.7501C>T	p.Gln2501X	*FLNC*	Nonsense	White/NH	1	31404	AD	LP
c.-6+2T>C	N/A	*GATA4*	Splice Site	White/NH	1	31376	AD	LP
c.433delC	p.Leu145fs	*GJA5*	Frameshift	White/NH	5	282120	AD	LP
c.508A>T	p.Lys170X	*HRAS*	Nonsense	White/NH	9	280744	AD	LP

© 2021 Yoneda ZT et al. *JAMA Cardiol.*

c.708-2A>C	N/A	*JUP*	Splice Site	White/NH	25	237434	AD	LP
c.708-2A>C	N/A	*JUP*	Splice Site	White/NH	25	237434	AD	LP
c.1282C>T	p.Gln428X	*KCNA5*	Nonsense	White/NH	8	282442	AD	LP
c.2200C>T	p.Arg734Cys	*KCNH2*	Missense	White/NH	2	280272	AD	LP
c.1400G>A	p.Arg467Glu	*KCNQ1*	Missense	White/NH	7	282350	AD	P
c.310C>T	p.Arg104Cys	*KCNQ1*	Missense	White/NH	1	31342	AD	P
c.457delG	p.Val153fs	*KCNQ1*	Frameshift	White/NH	---	---	AD	LP
c.19C>T	p.Pro7Ser	*KCNQ1*	Missense	White/NH	5	26538	AD	LP
c.415C>A	p.Leu139Met	*KCNQ1*	Missense	White/NH	1	31400	AD	LP
c.1234C>T	p.Arg412Trp	*KCNQ1*	Missense	White/NH	1	31382	AD	P
c.496C>T	p.Arg166Cys	*KCNQ1*	Missense	White/NH	10	281640	AD	LP
c.1605C>G	p.Tyr535X	*KCNQ1*	Nonsense	White/NH	12	209918	AD	LP
c.475G>T	p.Glu159X	*LMNA*	Nonsense	White/NH	1	31380	AD	P
c.647G>A	p.Arg216Cys	*LMNA*	Missense	White/NH	7	282556	AD	LP
c.290A>C	p.Lys97Thr	*LMNA*	Missense	White/NH	1	31390	AD	LP
c.1493G>A	p.Trp498X	*LMNA*	Nonsense	White/NH	1	31374	AD	LP
c.646C>T	p.Arg216Cys	*LMNA*	Missense	White/NH	2	282468	AD	P
c.48delC	p.Ser17fs	*LMNA*	Frameshift	White/NH	1	31354	AD	P
c.1315C>T	p.Arg439Cys	*LMNA*	Missense	White/NH	8	280234	AD	LP
c.1580G>A	p.Arg527His	*LMNA*	Missense	White/NH	10	250812	AD	P
c.1567G>A	p.Gly523Arg	*LMNA*	Missense	White/NH	1	31380	AD	LP
c.1576C>T	p.Arg526X	*MIB1*	Nonsense	White/NH	21	282742	AD	LP
c.2511_2514dupTGCT	p.Thr839fs	*MIB1*	Frameshift	White/NH	1	31378	AD	LP
c.2650_2651delAT	p.Met884fs	*MIB1*	Frameshift	White/NH	6	282560	AD	LP
c.1622delG	p.Gly541fs	*MIB1*	Frameshift	White/NH	1	31396	AD	LP
c.1504C>T	p.Arg502Trp	*MYBPC3*	Missense	White/NH	13	280632	AD	P
c.1624G>C	p.Glu542Gln	*MYBPC3*	Missense	White/NH	5	262678	AD	P
c.3330+2T>G	N/A	*MYBPC3*	Splice Site	White/NH	1	31314	AD	P
c.927-9G>A	N/A	*MYBPC3*	Splice Site	White/NH	1	31364	AD	P
c.3979-2A>C	N/A	*MYH6*	Splice Site	White/NH	240	139710	AD	LP
c.1299T>A	p.Tyr433X	*MYH6*	Nonsense	White/NH	1	31368	AD	LP
c.3979-2A>C	N/A	*MYH6*	Splice Site	White/NH	240	139710	AD	LP
c.3979-2A>C	N/A	*MYH6*	Splice Site	White/NH	240	139710	AD	LP
c.3979-2A>C	N/A	*MYH6*	Splice Site	White/NH	240	139710	AD	LP
c.3979-2A>C	N/A	*MYH6*	Splice Site	White/NH	240	139710	AD	LP

© 2021 Yoneda ZT et al. *JAMA Cardiol.*

c.3979-2A>C	N/A	*MYH6*	Splice Site	White/NH	240	139710	AD	LP
c.3979-2A>C	N/A	*MYH6*	Splice Site	White/NH	240	139710	AD	LP
c.3860-2A>G	N/A	*MYH6*	Splice Site	White/NH	1	31402	AD	LP
c.3979-2A>C	N/A	*MYH6*	Splice Site	White/NH	240	139710	AD	LP
c.1816G>A	p.Val606Met	*MYH7*	Missense	White/NH	2	282868	AD	P
c.5507C>T	p.Ser1836Leu	*MYH7*	Missense	White/NH	20	281784	AD	LP
c.3133C>T	p.Arg1045Cys	*MYH7*	Missense	White/NH	8	282850	AD	P
c.2155C>T	p.Arg719Trp	*MYH7*	Missense	White/NH	1	31394	AD	P
c.5507C>T	p.Ser1836Leu	*MYH7*	Missense	White/NH	20	281784	AD	LP
c.2722C>G	p.Leu908Val	*MYH7*	Missense	White/NH	1	31398	AD	P
c.610C>T	p.Arg204Cys	*MYH7*	Missense	White/NH	6	282876	AD	LP
c.611G>A	p.Arg204His	*MYH7*	Missense	White/NH	5	282888	AD	P
c.4817G>A	p.Arg1606His	*MYH7*	Missense	White/NH	11	282872	AD	LP
c.3133C>T	p.Arg1045Cys	*MYH7*	Missense	White/NH	8	282850	AD	P
c.1447G>A	p.Glu483Lys	*MYH7*	Missense	White/NH	2	282856	AD	P
c.1988G>A	p.Arg663His	*MYH7*	Missense	White/NH	4	282842	AD	P
c.1727A>G	p.His576Arg	*MYH7*	Missense	White/NH	5	282858	AD	LP
c.2221G>A	p.Gly741Arg	*MYH7*	Missense	White/NH	1	31394	AD	P
c.1988G>A	p.Arg663His	*MYH7*	Missense	White/NH	4	282842	AD	P
c.2644C>G	p.Gln882Glu	*MYH7*	Missense	White/NH	1	31378	AD	P
c.1366_1371dupTTCATA	p.Ile457_Gly458insPheIle	*MYH7*	Insertion/Deletion	White/NH	1	31404	AD	LP
c.5342G>A	p.Arg1781His	*MYH7*	Missense	White/NH	5	282896	AD	LP
c.427G>A	p.Glu143Lys	*MYL3*	Missense	White/Hispanic	4	35436	AD	LP
c.434T>C	p.Phe145Ser	*NKX2-5*	Missense	White/NH	1	31376	AD	LP
c.29T>C	p.Leu10Pro	*SCN3B*	Missense	White/NH	61	282860	AD	LP
c.29T>C	p.Leu10Pro	*SCN3B*	Missense	White/NH	61	282860	AD	LP
c.1936delC	p.Gln646fs	*SCN5A*	Frameshift	White/NH	1	31374	AD	P
c.1127G>A	p.Arg376His	*SCN5A*	Missense	White/NH	2	247596	AD	P
c.133_136delCTCT	p.Leu45fs	*SOS2*	Frameshift	White/NH	2	279448	AD	LP
c.451delC	p.Arg151fs	*TNNT2*	Frameshift	White/NH	14	281472	AD	LP
c.82G>C	p.Asp28His	*TPM1*	Missense	White/NH	1	1156	AD	LP
c.247dupG	p.Ala83fs	*TRPM4*	Frameshift	White/NH	13	265796	AD	LP
c.1000C>T	p.Arg334X	*TRPM4*	Nonsense	White/NH	5	281178	AD	LP
c.2295dupG	p.Arg766fs	*TRPM4*	Frameshift	White/NH	8	187350	AD	LP
c.424G>A	p.Val142Ile	*TTR*	Missense	Black/NH	405	24968	AD	P

© 2021 Yoneda ZT et al. *JAMA Cardiol.*

c.227A>G	p.His76Arg	*TTR*	Missense	White/NH	1	31406	AD	LP
c.659dupA	p.Asn220fs	*VCL*	Frameshift	White/NH	1	31248	AD	LP
c.2828_2829delCT	p.Pro943fs	*VCL*	Frameshift	Black/NH	12	24968	AD	LP

*****For non-white and Hispanic participants, ancestry-specific allele frequencies are reported. NH=non-Hispanic. AD=autosomal dominant. XLR=X-linked recessive.

© 2021 Yoneda ZT et al. *JAMA Cardiol.*

eTable 3. List of P/LP loss-of-function *TTN* variants

Variant (DNA)	Variant (protein)	Mutation Type	Race/ Ethnicity	Variant Allele Count gnomAD*	Total Allele Number gnomAD	Domain	Exon	PSI	ACMG Class
c.6555_6556insTGTAAGGAAACAGACA	p.Lys2186fs	Frameshift	White/NH	8	282064	I Band	30	100	LP
c.6555_6556insTGTAAGGAAACAGACA	p.Lys2186fs	Frameshift	White/NH	8	282064	I Band	30	100	LP
c.6555_6556insTGTAAGGAAACAGACA	p.Lys2186fs	Frameshift	White/NH	8	282064	I Band	30	100	LP
c.9610C>T	p.Arg3204X	Nonsense	White/NH	1	31386	I Band	42	100	LP
c.16529delT	p.Val5510fs	Frameshift	White/NH	1	31386	I Band	47	100	LP
c.11183dupG	p.Leu3729fs	Frameshift	White/NH	31	280174	I Band	47	100	LP
c.12575G>A	p.Trp4192X	Nonsense	White/NH	1	31362	I Band	47	100	LP
c.12587C>A	p.Ser4196X	Nonsense	White/NH	5	279924	I Band	49	100	LP
c.13859delG	p.Gly4620fs	Frameshift	White/NH	1	31360	I Band	49	100	LP
c.12587C>A	p.Ser4196X	Nonsense	White/NH	5	279924	I Band	49	100	LP
c.12587C>A	p.Ser4196X	Nonsense	White/NH	5	279924	I Band	49	100	LP
c.47494C>T	p.Arg15832X	Nonsense	White/NH	2	279366	A Band	254	100	P
c.48167delC	p.Pro16056fs	Frameshift	White/NH	1	31306	A Band	258	100	LP
c.53206C>T	p.Arg17736X	Nonsense	White/NH	1	31348	A Band	278	100	LP
c.57603C>A	p.Cys19201X	Nonsense	White/NH	3	273062	A Band	296	100	LP
c.59460G>A	p.Trp19820X	Nonsense	White/NH	1	31252	A Band	302	100	P
c.62424delC	p.Asp20808fs	Frameshift	White/NH	1	31366	A Band	305	100	LP
c.63370C>T	p.Gln21124X	Nonsense	White/NH	1	31344	A Band	306	100	LP
c.69421_69422insAAAAG	p.Gly23141fs	Frameshift	White/NH	1	31354	A Band	326	100	LP
c.86742_86745delCTAT	p.Tyr28915fs	Frameshift	White/NH	1	31410	A Band	327	100	LP
c.78178G>T	p.Glu26060X	Nonsense	White/NH	1	31394	A Band	327	100	P
c.77145dupC	p.Ser25716fs	Frameshift	White/NH	1	31382	A Band	327	100	P
c.70162C>T	p.Arg23388X	Nonsense	White/NH	1	31378	A Band	327	100	P
c.85090C>T	p.Arg28364X	Nonsense	White/NH	1	31368	A Band	327	100	P
c.89197+1G>C	N/A	Splice Site	White/NH	1	31408	A Band	334	100	LP
c.89265G>A	p.Trp29755X	Nonsense	White/NH	2	31396	A Band	335	100	LP
c.91918_91919delTG	p.Trp30640fs	Frameshift	White/NH	1	31400	A Band	339	100	LP
c.92317C>T	p.Arg30773X	Nonsense	White/NH	1	31392	A Band	340	100	P
c.92284_92288dupAAAAG	p.Ser30763fs	Frameshift	Black/NH	2	24188	A Band	340	100	P
c.92286_92287delAA	p.Ser30763fs	Frameshift	White/NH	1	31392	A Band	340	100	LP

© 2021 Yoneda ZT et al. *JAMA Cardiol.*

c.95082dupC	p.Gly31695fs	Frameshift	White/NH	1	31398	A Band	343	100	P
c.98299_98300delAG	p.Arg32767fs	Frameshift	White/NH	1	248686	A Band	353	100	P
c.99936G>A	p.Trp33312X	Nonsense	White/NH	2	31400	A Band	357	100	LP
c.99936G>A	p.Trp33312X	Nonsense	White/NH	2	31400	A Band	357	100	LP
c.100587G>A	p.Trp33529X	Nonsense	White/NH	2	31390	A Band	358	100	P
c.104867delG	p.Gly34956fs	Frameshift	White/NH	1	31396	M-Line	359	100	LP
c.107578C>T	p.Gln35860X	Nonsense	White/NH	4	280510	M-Line	363	99	P
c.107889delA	p.Lys35963fs	Frameshift	White/NH	2	280562	M-Line	364	100	P

*****For non-white and Hispanic participants, ancestry-specific allele frequencies are reported. NH=non-Hispanic.

© 2021 Yoneda ZT et al. *JAMA Cardiol.*

eTable 4. List of participants with 2 or more disease-associated variants

ID	Variant (DNA)	Variant (protein)	Gene	Mutation Type	Sex	Race/ Ethnicity	Variant Allele Count gnomAD	Total Allele Number gnomAD	Inheritance	ACMG Class
1257	c.646C>T	p.Arg216Cys	*LMNA*	Missense	M	White/NH	2	282468	AD	P
	c.12575G>A	p.Trp4192X	*TTN*	Nonsense	M	White/NH	1	31362	AD	LP
1373	c.1988G>A	p.Arg663His	*MYH7*	Missense	M	White/NH	4	282842	AD	P
	c.11183dupG	p.Leu3729fs	*TTN*	Frameshift	M	White/NH	31	280,174	AD	LP
1398	c.338C>T	p.Ser113Leu	*CPT2*	Missense	M	White/NH	393	282834	AD	P
	c.6555_6556insTGTAAGGAAACAGACA	p.Lys2186fs	*TTN*	Frameshift	M	White/NH	8	282064	AD	LP
1665	c.1605C>G	p.Tyr535X	*KCNQ1*	Nonsense	F	White/NH	12	209,918	AD	LP
	c.610C>T	p.Arg204Cys	*MYH7*	Missense	F	White/NH	6	282,876	AD	LP
1723	c.451delC	p.Arg151fs	*TNNT2*	Frameshift	M	White/NH	14	281,472	AD	LP
	c.6555_6556insTGTAAGGAAACAGACA	p.Lys2186fs	*TTN*	Frameshift	M	White/NH	8	282064	AD	LP
1766	c.3133C>T	p.Arg1045Cys	*MYH7*	Missense	M	White/NH	8	282,850	AD	P
	c.9610C>T	p.Arg3204X	*TTN*	Nonsense	M	White/NH	1	31,386	AD	LP
1825	c.1282C>T	p.Gln428X	*KCNA5*	Nonsense	M	White/NH	8	282,442	AD	LP
	c.48167delC	p.Pro16056fs	*TTN*	Frameshift	M	White/NH	1	31,306	AD	LP
1875	c.496C>T	p.Arg166Cys	*KCNQ1*	Missense	M	White/NH	10	281,640	AD	LP
	c.1624G>C	p.Glu542Gln	*MYBPC3*	Missense	M	White/NH	5	262,678	AD	P
	c.3979-2A>C	N/A	*MYH6*	Splice Site	M	White/NH	240	139710	AD	LP
2118	c.7977_7978delGA	p.Lys2660fs	*AKAP9*	Frameshift	M	White/NH	2	255474	AD	LP
	c.415-1G>T	N/A	*CSRP3*	Splice Site	M	White/NH	2	282724	AD	LP

NH=non-Hispanic. AD=autosomal dominant.

© 2021 Yoneda ZT et al. *JAMA Cardiol.*
